# Evaluating conceptual model measurement and psychometric properties of Oral health-related quality of life instruments available for older adults: a systematic review

**DOI:** 10.1186/s12955-023-02218-7

**Published:** 2024-01-13

**Authors:** Naira Figueiredo Deana, Yolanda Pardo, Montse Ferrer, Gerardo Espinoza-Espinoza, Olatz Garin, Patricia Muñoz-Millán, Claudia Atala-Acevedo, Àngels Pont, Margarita Cancino, Carlos Zaror

**Affiliations:** 1 Department of Pediatric Dentistry and Orthodontics, Faculty of Dentistry, Universidad de La Frontera, Temuco, Chile; 2 Center for Research in Epidemiology, Economics and Oral Public Health (CIEESPO), Faculty of Dentistry, Universidad de La Frontera, Temuco, Chile; 3 Doctoral Program in Morphological Sciences, Universidad de La Frontera, Temuco, Chile; 4 Universitat Autònoma de Barcelona, Barcelona, Spain; 5 Health Services Research Group, IMIM (Hospital del Mar Medical Research Institute), Barcelona, Spain; 6 CIBER Epidemiología y Salud Pública (CIBERESP), Barcelona, Spain; 7 Universitat Pompeu Fabra, Barcelona, Spain; 8 Department of Public Health, Faculty of Medicine, Universidad de La Frontera, Temuco, Chile; 9 Department of Psychology, Universidad de La Frontera, Temuco, Chile; 10 Laboratory of cognition, Aging and Health, Universidad de La Frontera, Temuco, Chile

**Keywords:** Oral health-related quality of life, Aged, EMPRO, Instruments, Psychometrics, Outcome assessment

## Abstract

**Background:**

Older adults present a variety of oral diseases and conditions, in addition to co-morbidities and limited access to dental care, which significantly impact their oral health-related quality of life (OHRQoL). There are many instruments published to measure OHRQoL. However, it is challenging for clinicians and researchers to choose the best instrument for a given purpose.

**Purpose:**

To identify OHRQoL instruments available for older adults and summarize the evidence on the conceptual and measurement model, psychometric properties, interpretability, and administration issues of OHRQoL instruments available for older adults through a systematic review.

**Methods:**

A systematic search was conducted in MEDLINE, EMBASE, LILACS, and CENTRAL up to February 2023. Articles reporting information on the concept model measurement, psychometric properties, and administration issues of an instrument measuring OHRQoL in older adults were included. Two researchers independently evaluated each instrument using the Evaluating Measures of Patient-Reported Outcomes (EMPRO) tool. The overall score and seven attribute-specific scores were calculated (range 0–100): Conceptual and measurement model, Reliability, Validity, Responsiveness, Interpretability, Burden, and Alternative forms.

**Results:**

We identified 14 instruments evaluated in 97 articles. The overall score varied between 73.7 and 8.9, with only six questionnaires over the threshold score 50.0. EORTC QLQ OH-15 (cancer-specific questionnaire) achieved the highest score (73.7), followed by OHIP (generic OHRQoL questionnaire) (66.9), GOHAI (generic OHRQoL questionnaire) (65.5), and OHIDL (generic OHRQoL questionnaire) (65.2). Overall, the Conceptual and measurement model and Validity showed the best performance, while Responsiveness and Interpretability showed the worst. Insufficient information was presented for an overall evaluation of DSQ and OHAI.

**Conclusion:**

The evidence supports using EORTC QLQ-OH15 as a specific instrument to assess OHRQoL in cancer patients and the OHIP-49, GOHAI, or OHIDL as generic instruments to assess OHRQoL either for cross-sectional or longitudinal studies in older adults.

**Supplementary Information:**

The online version contains supplementary material available at 10.1186/s12955-023-02218-7.

## Introduction

Today people tend to live for longer, however, the rate of aging of the population as a whole has accelerated [1]. The World Health Organization estimates that between 2015 and 2050, the percentage of the world’s population aged over 60 years will double from 12 to 22%; and that by 2030, one in six people in the world will be aged 60 or over [1]. A healthy old age is related with maintaining quality of life, allowing people to carry out their everyday activities normally [2].

Older adults present a wide variety of oral problems, such as caries, periodontal disease, tooth loss, non-functional dentures, lesions in the oral mucosa, and xerostomia, which directly affect their eating and nutrition habits [3, 4]. Extensive tooth loss may affect their speech, and chewing together with aesthetic implications, leading to problems with self-esteem and social interaction [5–8]*.* All the diseases and conditions mentioned above, in addition to co-morbidities and limited access to dental care in older adult populations, could significantly impact their quality of life [9, 10].

The concept of Oral Health-Related Quality of Life (OHRQoL) is conceived of as a multi-dimensional, self-reported evaluation to measure the impact of oral health on everyday activities [11]. In response to this need, various generic (Geriatric Oral Health Assessment Index-GOHAI, Oral Health Impact Profile-OHIP) and condition-specific instruments (Prosthetic Quality of Life-PQL, Oral Aesthetic-related quality of life-QoLDAS) have been developed to measure OHRQoL, however, according to our knowledge, there is no comparative evaluation of psychometric properties and applicability of OHRQoL instruments developed and validated for older adults. A comparative evaluation that identifies the strengths and weaknesses would facilitate the choice of the most suitable tool for clinical or research purposes to determine the expectations and perceptions about OHRQoL in this population. Therefore, unsuitable OHRQoL instruments for specific purposes or with deficient psychometric properties can introduce bias through unreliable effect estimates, leading to wrong clinical decisions. In addition, identifying suitable instruments to measure OHRQoL in older adults could contribute to formulating public policies that consider the user’s perspectives to improve their quality of life. Nevertheless, the absence of a valid and reliable OHRQoL measure could hinder this purpose.

This study aimed to identify OHRQoL instruments available for older adults and summarize the evidence on the conceptual and measurement model, psychometric properties, interpretability, and administration issues of OHRQoL instruments available for older adults through a systematic review.

## Material and methods

### Protocol

For this study, we used the methodology published previously [12]. We used the Preferred Reporting Items for Systematic Reviews and Meta-analysis (PRISMA) guidelines to report this systematic review [13–15] (Online Resource 1). This study was registered in PROSPERO (CRD42019133875).

### Eligibility criteria

Qualitative, observational and experimental studies reporting information on the conceptual and measurement model, the psychometric properties (reliability, validity and responsiveness), interpretability, and the administration (administration burden and alternative modes of administration) of OHRQoL instruments in older adults (> 60 years old or average age over 60 years) were included. Development studies for instruments that were not initially identified in the search were also included, regardless of the population’s age included. Articles written in English, Spanish, Portuguese, French, German and Italian were eligible, including studies both of original instruments and of versions validated for other countries.

Studies that did not evaluate the conceptual and measurement model, psychometric properties or administration of OHRQoL questionnaires, studies that evaluated instruments measuring patient-reported outcomes (PRO) other than the quality of life, and studies without information on the age of the participants were excluded.

### Information sources and search

A systematic search was conducted from inception to February 2023 in the following databases: MEDLINE, EMBASE, LILACS, and CENTRAL. The search strategy used in Medline is listed in the supplementary material (Online Resource 2).

It was complemented by a manual review of the references of the articles included and by online databases of PRO instruments: PROQOLID (https://eprovide.mapi-trust.org) and BiblioPRO (www.bibliopro.org).

### Study selection

Pairs of reviewers (CAA-GEE, PMM-CZ) in duplicate selected titles, abstracts, and full text. Any disagreement between the two review authors over the eligibility of a study was resolved through a third reviewer (YP).

### Data collection process

Each oral health-related quality of life instrument was evaluated independently by two reviewers with training and experience in measuring PRO (AP, CAA, CZ, GEE, MF, NFD, PMM, OG, or YP). The instruments were evaluated in the EMPRO online platform (https://empro.imim.es/es/principal). Disagreements on the criteria analysed were resolved by consensus between the evaluators.

### Evaluating measures of patient-reported outcomes

The EMPRO tool consists of 39 criteria assessing both the methodological quality of the included studies (11 criteria) and the results regarding their psychometric properties (13–16 criteria, since 3 could be assessed as not applicable), considering 8 attributes: 1.Conceptual and measurement model; 2.Reliability; 3.Validity; 4.Responsiveness; 5.Interpretability; 6.Burden (time, effort, and other demands on administrators and respondents); 7.Alternative modes of administration; 8.Cross-cultural and linguistic adaptation. The latter attribute was not completed in our case, because it was outside the scope of this study.

Agreement with each item is answered on a four-point Likert scale, from 4 (strongly agree) to 1 (strongly disagree), and there is also a “no information” option. Five items allow a reply of “not applicable”. Items for which the response option is “no information” are assigned a score of 1 (lowest possible score).

The overall score is constructed from the first five attributes. These attributes assess both the methodological quality of the included studies (11 criteria) and the results regarding their psychometric properties (13–16 criteria, since three could be assessed as not applicable) [12].

### Strategy for data synthesis

Attribute-specific scores and an overall score were calculated for each instrument. The mean score of the items was calculated for each attribute when at least 50% of the attributes were rated. Mean scores were linearly transformed into a range from 0 (worst possible score) to 100 (best possible score). Separate sub-scores for the Reliability and Burden attributes were calculated, as they are composed of two components each: “internal consistency” and “reproducibility” for Reliability and “respondent” and “administrative” for Burden. For Reliability, as the two components represent different approaches to examine the same attribute, the higher sub-score was chosen. For Burden, the final score was calculated as their mean as the two components assess different aspects of the same attribute.

The overall score was computed by calculating the mean of the five metric-related attributes: Conceptual and measurement model, Reliability, Validity, Responsiveness, and Interpretability. The overall score was only calculated when at least three of these five attributes had a score. EMPRO scores were considered acceptable if they reached at least 50 points (half the theoretical maximum of 100 points) [12, 16].

## Results

### Search results

**Fig. 1:**
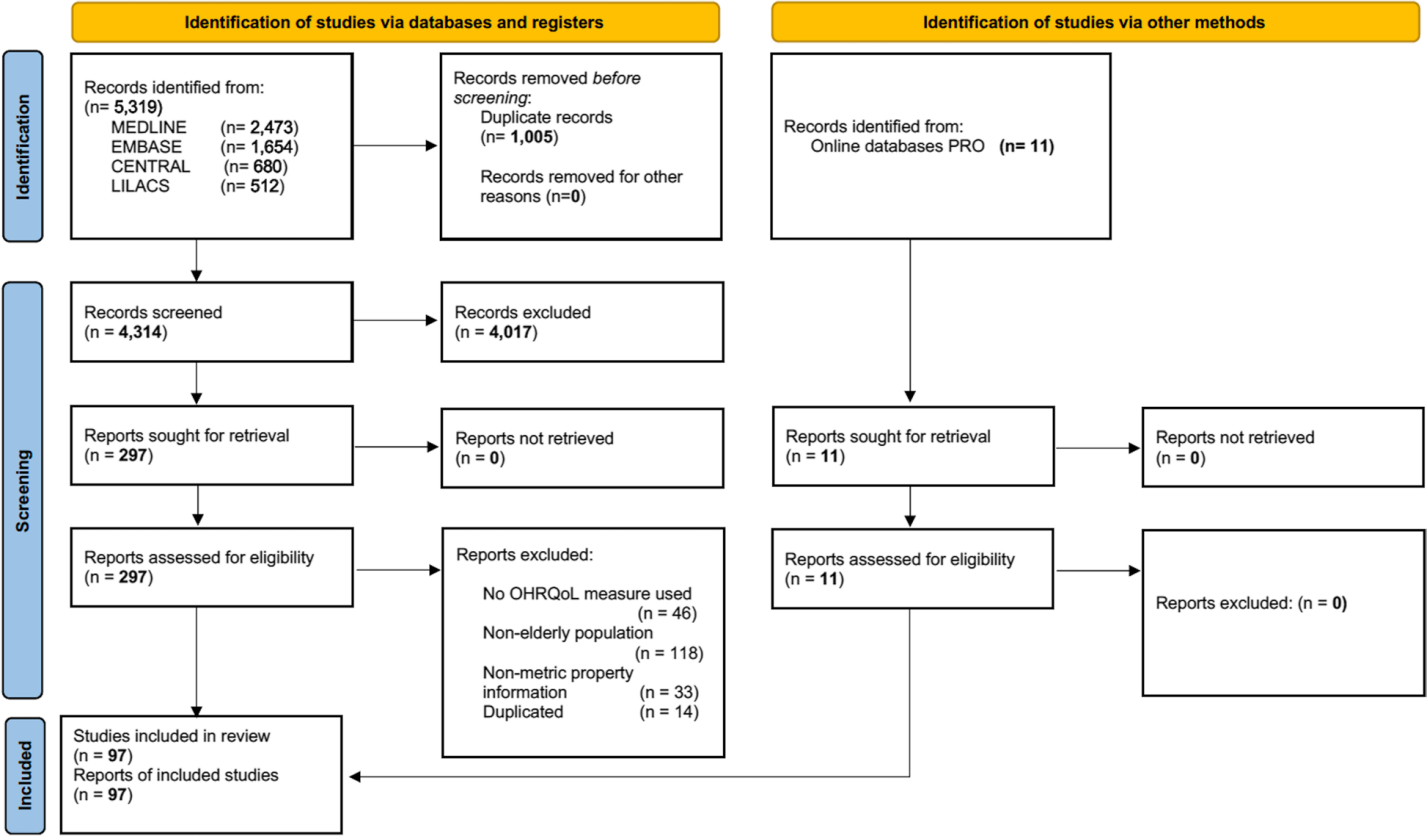
Flow-chart of the studies and reports included

The search identified 5319 references (Fig. 1). After excluding 1005 duplicates and reviewing the titles, abstracts and full-text, 297 articles were selected. Of these, 211 were excluded, and 86 studies were selected as potentially relevant for data extraction. Twelve further articles were identified by manual search and from online databases of PRO. Thus, a total of 97 full-text articles assessed 14 instruments were considered in the EMPRO evaluation (see characteristics of included in Online Resource 3). The number of articles found per instrument ranged from 1 to 43, with five articles providing information for more than one instrument.

### Characteristics of instruments

Table 1 shows the characteristics of the instruments identified. The instruments identified were developed between 1993 and 2020. Seven instruments were developed in English, three in Spanish, and one in different languages (British, English, Dutch, French, German, Greek, Hebrew, Italian, Polish, Swedish, and Norwegian). The Geriatric Oral Health Assessment Index (GOHAI), The Oral Health Impact Profile (OHIP), and The Oral Hygiene Assessment Instrument (OHAI) were the only instruments adapted to other languages. The European Organization of Research and Treatment of Cancer, Oral Health Module (EORTC QLQ-OH-15) was developed in different countries and languages. Most instruments are self-administered (9/14), while five were developed for administration in an interview. Seven instruments were developed exclusively for an older adult population (DSQ, GOHAI, IPQ-RDE, OHAI, OHIDL, OHQoL-UK-W, OHIP); three for adult and older adult populations (EORTC QLQ-OH15, QoLDAS-9: Oral Aesthetic-related quality of life, PQL: Prosthetic Quality of Life, QoLIP-10:The Quality of Life with Implant-Protheses); and three were developed for an adult population but were subsequently validated for older adult populations (LORQ: Liverpool Oral Rehabilitation Questionnaire, OIDP: Oral Impacts on Daily Performance, OHRQL: Oral Health-Related Quality of Life). The majority of the instruments (8/14) were generic for measuring OHRQoL, and only six were designed to assess specific treatments and health conditions related with oral health. Within the specific instruments, DSQ was designed to measure patient satisfaction before and after prosthesis treatment. EORTC QLQ-OH15 focused on oral health and related QoL issues in all cancer diagnoses. LORQ is a specific questionnaire for head and neck cancer. PQL evaluates OHRQoL in individuals who use a removable prosthesis. QoLDAS-9 evaluates the quality of life-related with oral aesthetics in patients with restoration by prosthesis. Finally, QoLIP-10 evaluates the OHRQoL of patients who have received oral rehabilitation with Implant-Prostheses.

**Table 1:** Summarized characteristics of instruments designed or validated for old adults, in alphabetical order

Instrument	Country of development	Purpose of development (type of instrument)	Administration mode	Dimensions (no. of items)	Response options	Score (range)	Original and adopted languages	Number of studies evaluated
DSQ	NI	Edentulous patient (Specific)	Self-administered	General satisfaction, retention, comfort, stability, appearance, ability to speak, and occlusion (12)	Likert-scale	NI	Maltese	1
EORTC QLQ-OH15	10 countries: France, Germany, Greece, Israel, Italy, Netherlands, Norway, Poland, Sweden, UK	Oral health in cancer patients (Specific)	Interview administered	OH-QoL scale (8), 3 single items (sticky saliva/mouth soreness/ sensitivity to food/drink), 2 two-item contingency scales regarding use (yes/no) and problems with dentures and reception of (yes/no) and satisfaction with information	4-point Likert scale	Global score (0–100)	Dutch English French German Greek Hebrew Norwegian Polish Sinhalese Swedish	3
GOHAI	United States	Oral Health (Generic)	Self-administered	Physical function (4); Psychosocial function (5); pain or discomfort (3)	5-point Likert scale	Global score (12 to 60)	English Arabic Chinese Dutch German Greek Hindi Japanese Lebanese Malay Maltese Mexican Mandarin Chinese Nepalese Persian Portuguese Serbian Swedish Turkish Urdu	33
IPQ-RDE	US	Oral Health (Generic)	Interview administered	Identity (2); Timeline (5); Consequences (6); Control (6); Illness coherence (2); Treatment burden (5), Prioritization (3); Causal relationship (3); Activity restriction (3); Emotional representation (5)	5-points Likert scale	NI	English	1
LORQ	England	Head and neck cancer (Specific)	Self-administered	Oral function (12) denture satisfaction (13)	4-point Likert-scale	Global score (25 to 100)	English	2
OIDP	Thailand	Oral health (Generic)	Self-administered	Eating and enjoying food (1) Speaking and pronouncing clearly (1); Cleaning teeth (1); Sleeping and relaxing (1); Smiling, laughing and showing teeth without embarrassment (1); Maintain usual emotional state without being irritable (1); Carrying out major work or social role (1); Enjoying contact with people (1)	Frequency Score (0–5); Severity score (0–5)	Global Score (0 to 200)	Thai English Greek Japanese Mangalese Portuguese	10
OHAI	Sweden	Oral Health (Generic)	Interview administered	Background, social context (8) (Part I); Dental care and xerostomia (10) (Part I); Clinical examination (6) (Part II); Observation ADL (8) (Part III)	Qualitative evaluation (Parts I and II); Observational ADL part (part III) three response alternatives	Global Score Observational ADL part (11 to 33)	Sweden	1
OHIDL	Hong Kong	Oral health (Generic)	Semi-structured interviews administered	Part I: checklist of oral health problems and symptoms; Part II: Cleasing (1) Eating (6) Speaking (1) Appearance (2) Social (2) Psychological (2) Health (2) Finance (1): Part III: five global questions	Part II: 5-point Likert Scale	NI	Chinese	3
OHIP	United States	Oral health (Generic)	Self-administered	Functional limitation (9), Physical pain/discomfort (9), Psychological discomfort (5); Physical disability (9); Psychological disability (6); Social disability (5), Handicap (6)	5-point Likert scale	Global score (0 to 245)	English Albanian Arabic Australian Chinese Croatian Czech German Greek Hungarian Italian Japanese Korean Lebanese Maltese Mexican Nepalese Persian Polish Portuguese Romanian Serbian Sinhalese Spanish Swedish	44
OHQoL-UK-W	United Kingdon	Oral health (Generic)	Interview administered	Physical aspects (6) Social aspects (5) Psychological aspects (5)	Scale from 1 to 9	Global score (16 to 144)	English	1
OHRQL	United States	Oral health (Generic)	Self-administered	Symptom status: Pain (6); Dry mouth symptom (3). Function status: Eating/Chewing Function (3); Speech function (3); Social function (4); Psychological function (5). Health perceptions: Oral health perception (2)	5-point Likert-scale	NI	English	1
PQL	Spain	Total or partial removable prostheses (Specific)	Self-administered	Prosthetic fit (1), Chewing (1), Foreign body (1), Aesthetics (1), Communication (1), Realism of prosthesis (1), Unnoticeability (1), Hygiene (1), Food impaction (1), Functional comfort (1), Self-confidence (1)	5-point Likert-scale	Global score (11 to 55)	Spanish	1
QoLDAS-9	Spain	Dental aesthetics - prosthetically restored patients (Specific)	Self-administered	Psychofacial aesthetic (3) Interactive aesthetic (3) Socio-dental aesthetic (3)	Likert scale: −2 to + 2	- Global score (−18 to + 18)	Spanish	1
QoLIP-10	Spain	Patients wearing implant overdentures and hybrid prostheses (Specific)	Self-administered	Biopsychosocial dimension (5) Dental–facial aesthetics dimension (3) Performance dimension (2)	Likert scale: −2 to + 2	Global score (− 20 to + 20)	Spanish	2

*ADL* Activities of daily living, *NI *No information, *DSQ *Denture Satisfaction Questionnaire, *EORTC QLQ OH-15 *European organization of Research and Treatment of Cancer, Oral Health Module, *GOHAI* Geriatric Oral Health Assessment Index, *IPQ-RDE *Illness Perception Questionnaire Revised for Dental Use in Older/Elder Adults, *LORQ *Liverpool Oral Rehabilitation Questionnaire, *OIDP *Oral Impacts on Daily Performance, *OHAI *The Oral Hygiene Assessment Instrument, *OHIDL *Oral Health Impact on Daily Living, *OHIP *Oral Health Impact Profile, *OHRQL *Oral Health Related of Quality of Life, *OHQoL-UK-W *Oral Health Related of Quality of Life - UK, *PQL *Prosthetic Quality of Life, *QoLDAS-9* Oral Aesthetic-related quality of life, *QoLIP-10* The Quality of Life with Implant-Protheses

### Results of the EMPRO ratings

**Fig. 2:**
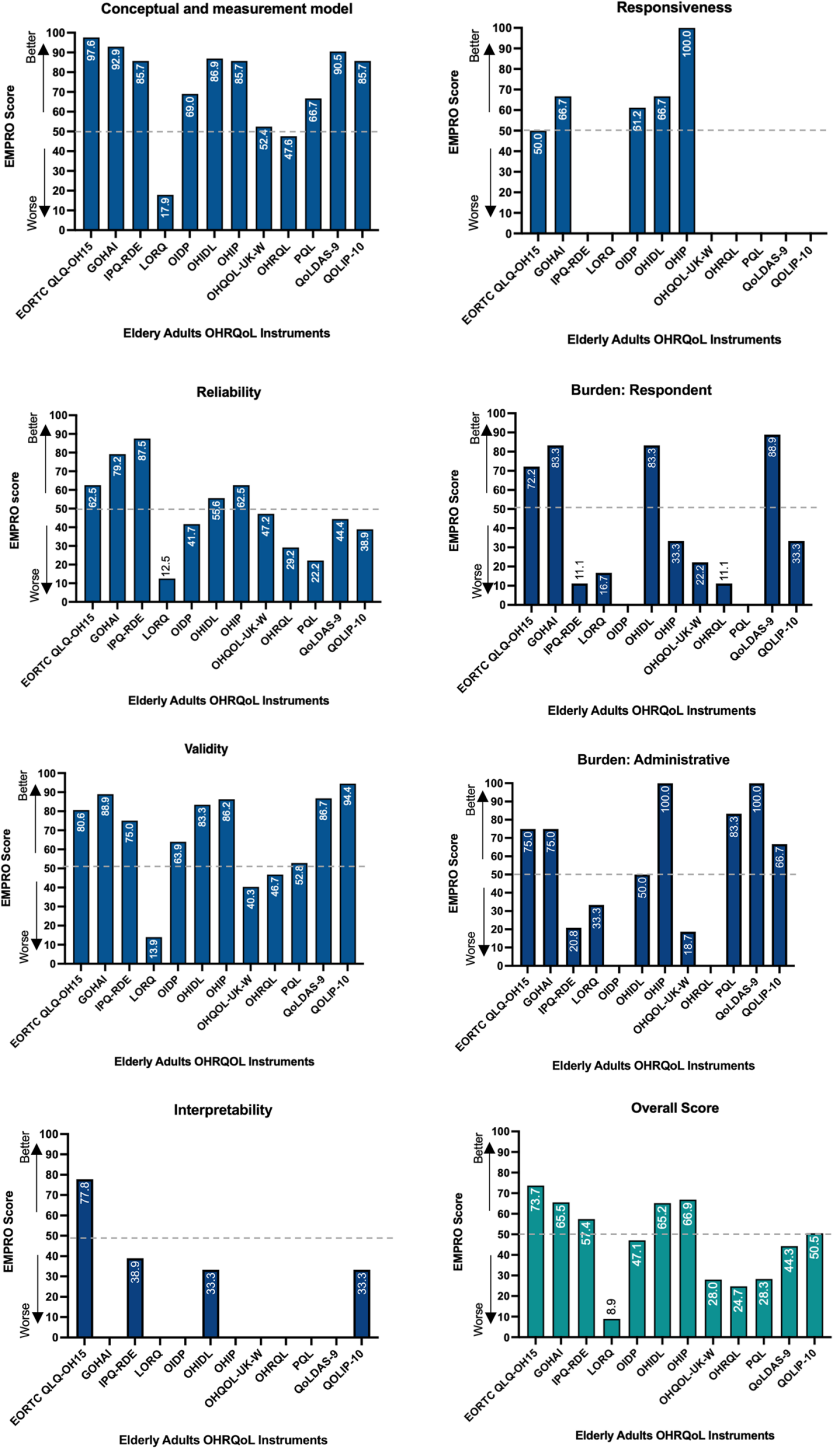
Overall EMPRO ranking and attribute-specific scores of instruments designed for the elderly (age > 65 years). The grey line on 50 (half of the theoretical maximum of 100 points) represents the reasonably acceptable cut-off defined for EMPRO scores. EORTC QLQ-OH-15: European organization of Research and Treatment of Cancer, Oral Health Module; GOHAI: Geriatric Oral Health Assessment Index; IPQ-RDE: Illness Perception Questionnaire Revised for Dental Use in Older/Elder Adults; LORQ: Liverpool Oral Rehabilitation Questionnaire; OIDP: Oral Impacts on Daily Performance; OHIDL: Oral Health Impact on Daily Living; OHIP: Oral Health Impact Profile; OHRQL: Oral Health Related of Quality of Life; OHQoL-UK-W: Oral Health Related of Quality of Life – UK; PQL: Prosthetic Quality of Life; QoLDAS-9: Oral Aesthetic-related quality of life; QoLIP-10: The Quality of Life with Implant-Protheses

The attribute Conceptual and measurement model presented the best performance, with 10/14 instruments obtaining a score higher than 50.0. The thresholds for this attribute varied between 17.9 and 97.6, with 3/14 instruments obtaining a score higher than 90 (EORTC QLQ-OH15, GOHAI, and QOLDAS-9). The OHAI obtained a score of 63.1 and the DSQ could not be evaluated as there was insufficient information for most aspects analysed in this attribute (Fig. 2). The OHAI and the DSQ were not included in the figures since they had insufficient information for an overall evaluation.

The thresholds for Reliability varied between 12.5 and 87.5. Five instruments had a score equal to or higher than 50.0; IPQ-RDE obtained the highest score, followed by GOHAI, OHIP, EORTC-OH15 and OHIDL. EMPRO score could not be obtained for DSQ, LORQ, and OHAI due to the lack of enough evidence identified (Fig. 2).

Validity was the attribute with the second-best performance in the instruments, with 9/14 instruments obtaining a score higher than 50.0. The thresholds varied between 13.9 for LORQ and 94.4 for QOLIP-10. DSQ and OHAI did not present sufficient information to assess this attribute (Fig. 2).

Interpretability presented the worst performance. Only four instruments presented sufficient information for evaluation, with scores of 77.8 for EORTC QLQ-OH15, 38.9 for IPQ-RDE and 33.3 for OHIDL and QOLIP-10 (Fig. 2).

Only five instruments presented sufficient information for evaluation of Responsiveness, all with scores over 50.0: OHIP rated the maximum score (100.0), GOHAI and OHIDL 66.7, OIDP 61.2, and EORTC QLQ-OH15 rated 50.0.

In evaluating the ease of use of the instruments, QoLDAS-9, OHAI and GOHAI obtained the highest scores for Respondent burden (88.9, 83.3 and 83.3). These instruments described the skills and time needed to complete the instrument, its acceptability, and the circumstances in which it is unsuitable for the respondent. The instruments which obtained the highest scores for questionnaire administration and scoring were QoLDAS-9 and OHIP, with 100.0 each. The high scores were because the instrument details the resources needed for the administration, the score calculation method is well described, and the associated burden is acceptable.

OHIP was the only instrument with alternative administration forms, in this case, application by an interview. Abbreviated versions of the original format of the instrument (OHIP-49) were also evaluated, namely OHIP-14 and OHIP-7. A specific version for edentulous patients has also been created (OHIP-Edent).

The instrument with the highest overall score was EORTC QLQ-OH15 with 73.7, followed by OHIP with 66.9, GOHAI with 65.5, and OHIDL with 65.2. The instruments with the lowest scores were OHRQL with 24.7 and LORQ, with 8.9. Six instruments obtained an overall score lower than 50.0 (LORQ, OIDP, OHQOL-UK-W, OHRQoL, PQL, QOLDAS-9). The overall scores for DSQ and OHAI were not analysed as they did not present information for at least 4 attributes evaluated by EMPRO (Fig. 2).

The detailed results of EMPRO for any specific criterion and attribute are shown in Table 2.

**Table 2:** Rating of each EMPRO items and attribute for OHRQoL in Elderly

	ATTRIBUTES	DSQ	EORTC QLQ-OH15	GOHAI	IPQ-RDE	LORQ	OIDP	OHAI	OHIDL	OHIP	OHQoL-UK-W	ORHQL	PQL	QoLDAS-9	QoLIP-10
	**CONCEPT AND MEASUREMENT MODE**L	NI	97.6	92.9	85.7	17.9	69.0	63.1	86.9	85.7	**52.4**	**47.6**	**66.7**	**90.5**	**85.7**
1	concept of measurement stated	+	++++	++++	++++	++	++++	++++	++++	++++	+++	++++	++++	++++	++++
2	obtaining and combining items described	–	++++	++++	++++	+	++++	++++	++++	+++	+++	+++	++++	++++	++++
3	rationality for dimensionality and scales	–	++++	+++	++++	+	++	++++	++++	++	++	+++	+++	++++	+++
4	involvement of target population	–	++++	+++	+++	+	++++	+++	+++	+++	++++	+++	++++	++++	+++
5	scale variability described and adequate	+	++++	+++	++	++	+	–	+++	++++	++	–	+	++	+++
6	level of measurement described	+	++++	++++	+++	++	++	++	+++	++++	++	+	–	++++	++++
7	procedures for deriving scores	–	+++	++++	++++	–	++++	–	++++	++++	++	–	+++	++++	+++
	**RELIABILITY - total score**	**–**	**62.5**	**79.2**	**87.5**	**12.5**	**41.7**	**–**	**55.6**	**62.5**	**47.2**	**29.2**	**22.2**	**44.4**	**38.9**
	*Reliability: internal consistency*														
8	data collection methods described	+	++++	++++	++++	+	++	–	++++	++++	++	++	++	+++	+++
9	Cronbach’s alpha adequate	+	+++	+++	+++	+	++	–	+++	+++	++++	+++	++	+++	++
10	IRT estimates provided	–	–	+++	+++	–	–	–	–	–	–	–	–	–	–
11	testing in different populations	n.a.	–	++++	++++	+	n.a.	–	n.a.	+++	n.a.	–	n.a.	n.a.	n.a.
	*Reliability: reproducibility*											–			
12	data collection methods described	+	+++	+++	–	+	++	–	–	+++	–	–	–	–	–
13	test-retest and time interval adequate	+	++++	++++	–	+	+++	–	–	+++	–	–	–	–	–
14	reproducibility coefficients adequate	+	+++	+++	–	+	++	–	–	++++	–	–	–	–	–
15	IRT estimates provided	+	–	–	–	–	–	–	–	–	–	–	–	–	–
	**VALIDITY**	**–**	**80.6**	**88.9**	**75.0**	**13.9**	**63.9**	**–**	**83.3**	**86.2**	**40.3**	**46.7**	**52.8**	**86.7**	**94.4**
16	content validity adequate	–	++++	++++	+++	–	+++	++++	+++	+++	++	++	++++	++++	++++
17	construct/criterion validity adequate	–	+++	+++	++++	++	++++	–	++++	+++	+++	++	++	+++	+++
18	sample composition described	–	++++	++++	+++	+	–	–	++++	++++	+++	+++	++++	++++	++++
19	prior hypothesis stated	–	+++	++++	+++	+	+++	–	++	+++	+++	+	++	+++	++++
20	rational for criterion validity	n.a.	++	+++	–	+	++++	–	n.a.	++++	–	n.a.	++	++++	++++
21	tested in different populations	–	++++	++++	++++	++	++	n.a.	n.a.	++++	–	+++	+	n.a.	++++
	**RESPONSIVENESS**	**–**	**50.0**	**66.7**	**–**	**–**	**61.2**	**–**	**66.7**	**100**	**–**	**–**	**–**	**–**	**–**
22	adequacy of methods	–	+++	+++	–	–	+	–	++++	++++	–	–	–	–	–
23	description of estimated magnitude of change	–	+++	+++	–	–	+++	–	++++	++++	–	–	–	–	–
24	comparison of stable and unstable groups	–	+	+++	–	–	++++	–	–	++++	–	–	–	–	–
	**INTERPRETABILITY**	**–**	**77.8**	**–**	**38.9**	**–**	**–**	**–**	**33.3**	**–**	**–**	**–**	**–**	**–**	**33.3**
25	rational of external criteria	–	++++	–	+++	–	–	–	+++	++++	–	–	–	–	–
26	description of interpretation strategies	–	++	–	++	–	–	–	++	–	–	–	–	–	++
27	how data should be reported stated	–	++++	–	+	–	–	–	–	–	–	–	–	–	+++
	**OVERALL SCORE**	**–**	**73.7**	**65.5**	**57.4**	**8.9**	**47.1**	**–**	**65.2**	**66.9**	**28.0**	**24.7**	**28.3**	**44.3**	**50.5**
	**BURDEN**														
	***Burden: respondent***	**–**	**72.2**	**83.3**	**11.1**	**16.7**	**–**	**83.3**	**–**	**33.3**	**22.2**	**11.1**	**–**	**88.9**	**33.3**
28	skills and time needed	–	+++	+++	++	+	–	++++	++	+++	+++	–	–	+++	++
29	impact on respondents	+++	+++	+++	+	+	+	+++	–	++++	+	++	+	++++	++++
30	not suitable circumstances	++	+++	++++	+	+	–	+++	–	–	+	+	–	++++	–
	***Burden: administrative***	**–**	**75.0**	**75.0**	**20.8**	**33.3**	**–**	**75.0**	**50.0**	**100.0**	**18.7**	**–**	**83.3**	**100.0**	**66.7**
31	resources required	–	+++	++++	+	+++	–	++++	++++	++++	+	–	+++	++++	++++
32	time required	n.a.	++++	++	+++	n.a.	–	++++	–	n.a.	+	n.a.	n.a.	n.a.	+++
33	training and expertise needed	n.a.	++	+++	+	n.a.	–	++	–	n.a.	++	n.a.	n.a.	n.a.	–
34	burden of score calculation	–	+++	++++	+	–	++	+++	++++	++++	++	–	++++	++++	++++
	**ALTERNATIVE MODES OF ADMINISTRATION**	–	–	–	–	–	–	–	–	**100.00**	–	–	–	–	–
35	metric characteristics	–		–	–	–	–	–	–	++++	–	–	–	–	–
36	comparability	–	–	–	–	–	–	–	–	++++	–	–	–	–	–

Explanation: ++++ 4 (strongly agree); +++ 3; ++ 2; + 1 (strongly disagree); − no information; n.a. not applicable. The higher the agreement the better the rating. Rows in white show EMPRO criteria assessing the results of the corresponding metric property, while rows in grey show EMPRO criteria assessing the methods applied to evaluate the corresponding metric property

*DSQ* Denture Satisfaction Questionnaire, *EORTC QLQ OH-15 *European organization for Research and Treatment of Cancer, Oral Health Module, *GOHAI *Geriatric Oral Health Assessment Index, *IPQ-RDE *Illness Perception Questionnaire Revised for Dental Use in Older/Elder Adults, *LORQ *Liverpool Oral Rehabilitation Questionnaire, *OIDP *Oral Impacts on Daily Performance, *OHAI *The Oral Hygiene Assessment Instrument, *OHIDL *Oral Health Impact on Daily Living, *OHIP *Oral Health Impact Profile, *ORHQL *Oral Health Related of Quality of Life, *OHQoL-UK-W *Oral Health Related of Quality of Life - UK, *PQL *Prosthetic Quality of Life, *QoLDAS-9 *Oral Aesthetic-related quality of life, *QoLIP-10* The Quality of Life with Implant-Protheses

## Discussion

Evaluation of OHRQoL plays an important role in clinical practice. As a result, several instruments have been developed to evaluate functional, social and psychological aspects of oral diseases or conditions disorder [17]. In this study, we identified and evaluated 14 instruments designed to measure OHRQoL in older adults. Of these, only six overcame the minimum score in EMPRO (50.0) for their administration in older patients to be recommended (EORTC QLQ-OH-15, GOHAI, IPQ-RDE, OHIDL, OHIP, QOLIP-10). EORTC QLQ-OH-15 was the instrument that obtained the best evaluation by the experts, followed by OHIP, GOHAI, and OHIDL.

EORTC QLQ-OH-15 is a supplementary module of the EORTC QLQ-C30 for assessing OHRQOL in cancer patients, addressing aspects such as pain, sensitivity to food and drink, saliva, information received, and use of dentures [18, 19]. It was developed for the adult and older adult populations, and it has been validated for different populations and languages.

OHIP, GOHAI, and OHIDL are generic instruments for evaluating OHRQoL in patients with oral diseases [2, 17]. Applying OHIP may involve a greater respondent burden than GOHAI, so a shorter version of the instrument, such as OHIP-14 or OHIP-EDENT, is a possible option. However, shorter versions of OHIP place more weight on psychological or behavioural aspects, while GOHAI prioritises aspects related to functional limitations and pain [17]. Previous studies have compared the psychometric properties of GOHAI and OHIP-14 for the older adult population. It was found that both instruments are suitable for evaluating the impact of oral pathologies on OHRQoL; however, GOHAI is better than the short forms of OHIP at detecting problems in oral function [17, 20].

El IPQ-RDE, a generic instrument for detecting single and multiple dental conditions in older adults [21]. It measures different aspects from those measured in EORTC QLQ-OH15, OHIDL, GOHAI and OHIP, such as the chronology of the disease, control of the symptoms, treatment burden and prioritisation of the disease. IPQ-RDE is a promising instrument, and it is probable that when new evidence is available, with more studies and improvements in some of its attributes, this instrument will prove to be an excellent option for measuring OHRQoL in older adults.

The majority of the instruments for evaluating OHRQoL in older adults are not suitable for detecting changes in oral health since Responsiveness was measured by five instruments (EORTC QLQ-OH-15, GOHAI, OHIDL, OHIP and OIDP). OHIP showed the best performance for Responsiveness, followed by GOHAI and OHIDL, making them recommended for longitudinal studies and clinical trials. Responsiveness is essential for ensuring that the changes reported are real and not the result of measurement errors. OIDP also obtained a good score for Responsiveness; however, it had poor internal consistency and inadequate coefficients of Reproducibility, which may affect the data in instruments used for longitudinal studies. OIDP is a generic, self-administered instrument translated into five languages other than the original. It evaluates serious oral impacts on daily performance [22]. The evaluation of OIDP could only be improved by developing strategies to make score interpretation easier, to describe the burden (respondent and administrative) and to increase internal consistency and reproducibility.

A generic instrument can detect the impact of oral or orofacial diseases, allowing comparisons of diseases and conditions [17]. On the other hand, generic instruments may be less sensitive, specific or useful for evaluating a specific disease [17]. Previous studies have shown that the EMPRO score is higher for generic than for specific instruments [23], very similar to what was found in our study. Evaluation by experts showed that only two (EORTC QLQ-OH-15 and QoLIP-10) of the six specific instruments obtained a score higher than 50.0. The EORTC QLQ-OH-15 showed the highest overall score and good performance in most domains; however, generic instruments such as the GOHAI, OHIDL and OHIP showed better performance in domains such as reliability and validity.

Evaluation by the EMPRO tool is based on the quantity and quality of the evidence published for each instrument. The absence of information for some attributes in EMPRO evaluation penalises the scores since the missing information is given the lowest possible score [23]. One factor which could have affected the performance of these instruments is the fact that only one or two studies per instrument were evaluated, with poor or missing information for some attributes.

The overall score was not calculated for DSQ and OHAI, as information was missing for at least half of the attributes evaluated by EMPRO. In the case of DSQ, not only was there no information for many attributes, but those evaluated obtained very low scores. All aspects of this instrument need to be improved. OHAI obtained a good score for Conceptual and measurement model (score = 63.1) and ease of use (respondent burden: 83.3; administrative burden: 75.0); however, there were insufficient data for evaluation of Reliability, Validity, Interpretability and Responsiveness.

Apart from EORTC QLQ-OH15, IPQ-RDE, OHAI and OHQoL-UK-W, all the instruments were developed for self-administration. The mode of administration may influence the quality of the data, and the way in which older adults answer the instrument. Self-administered instruments may require greater physical and cognitive capabilities in the respondents [24]. This reflects the need for the clinician/investigator to consider the patient’s condition before selecting the most appropriate instrument for evaluating OHRQoL in the older adult population.

### Strengths and limitations

The main strength of this study is that we also include instruments not explicitly developed for older adults but are currently used by clinicians and researchers in this population. Not including them would introduce a selection bias excluding valuable information on the validity, reliability and responsiveness of these instruments currently in use in this population.

The use of EMPRO is another strength of our study since it is designed to evaluate the performance of an instrument based on what is reported by all the studies that assessed a specific health problem. EMPRO has been shown to have high internal consistency, inter-rater agreement, and positive associations consistent with a priori hypotheses between EMPRO attribute scores and bibliometric quality indicators. In addition, according to the FDA (US Food and Drug Administration) guideline for patient-reported outcome measures [25], it is essential that the reliability, validity, sensitivity to change and the choice of interpretation method of an instrument be evaluated before use in the measurement of treatment benefit or risk in medical product clinical trials; all these properties are assessed attributes in EMPRO.

Our study presents certain limitations attributable to a variety of reasons. First, it is possible that we did not identify all the instruments of OHRQoL in older adults. To minimise this risk, we used a sensitive search strategy complemented by a manual search of the references and two online databases of PRO, as well as a duplicated review process. In addition, our systematic review has a limitation regarding language restrictions. We attempted to include research in various languages, including English, Spanish, Portuguese, French, German, and Italian. However, it is possible that some studies in other languages were not included in our inclusion criteria, introducing selection bias. Furthermore, the development instruments were included regardless of the age range of the participants in order to identify all the available information. Second, the cut-off point established as the threshold for considering EMPRO scores acceptable is questionable. This threshold was obtained with data from the first two EMPRO studies [12, 16]: the area under the receiver operating characteristic (ROC) curve evaluating the agreement between EMPRO attribute scores and the reviewers’ global recommendations was of 0.87 (data not shown but available upon request) and should be used only as a guideline for identifying gaps in the instruments. Third, the EMPRO evaluations may be biased by the individual experience of the evaluators; however, the evaluations were carried out by researchers with experience in the evaluation of PROMs, and at least one of the two evaluators belonged to the team that manages the EMPRO tool, minimizing this bias. Fourth, it is also important to bear in mind that the EMPRO criteria assess both the methodological quality of the studies and the results of the instrument metric properties, so there could be a risk that studies with adequate methodologies and poor results may obtain EMPRO scores above 50. However, to mitigate this potential risk, there are more EMPRO criteria focused on results than on methodological characteristics: 5 vs 2 in the conceptual and measurement model, 2–3 vs 1 for internal consistency, 2 vs 2 for reproducibility, 2–4 vs 2 for validity, 2 vs 1 for responsiveness, and 2 vs 1 for interpretability. Furthermore, in our EMPRO evaluation, all instruments with scores over 50 also have a good rating in the results criteria. Fifth, EMPRO global score is a summary of the five metric attributes assessed that facilitates a synthesis, but it is recommended to consider scores of each of these five attributes separately according to the purpose for applying the instrument. Sixth, because the EMPRO tool is based on the quantity and quality of the evidence published for each instrument, instruments developed recently, for which little evidence is available, may have been penalised. On the other hand, no overall score was calculated for instruments which did not present information for at least half of the attributes, in order not to penalise them too heavily for lack of information. Finally, we didn’t perform a meta-analysis since EMPRO makes a qualitative evaluation by experts with a consensus process of each OHRQoL instrument considering the variability of the data reported in the different studies to make a judgment and not just the average as would be the case with meta-analysis. In addition, the variability between studies related to the characteristics of the population and methods used to measure the different psychometric properties could generate a significant heterogeneity affecting the certainty estimate obtained with meta-analysis.

## Conclusions

The evidence supports using EORTC QLQ-OH15, as a specific instrument to assess OHRQoL in cancer patients and the OHIP-49, GOHAI, or OHIDL, as generic instruments to assess OHRQoL either for cross-sectional or longitudinal studies in older adults. Future studies of the other instruments should focus on attributes such as Burden, Interpretability and Responsiveness, in order to re-evaluate their usefulness in this population. Our results will facilitate decision-making by clinicians and investigators in choosing the best instrument according to the needs and requirements of older adults.

### Supplementary Information

**Additional file 1.**

**Additional file 2.**

**Additional file 3.**

## Data Availability

The datasets generated during and/or analysed during the current study are available from the corresponding author on reasonable request.

## Abbreviations

OHRQoL: oral health-related quality of life
OHRQL: Oral Health-Related Quality of Life
EMPRO: Evaluating Measures of Patient-Reported Outcomes
EORTC QLQ OH-15: European Organization for Research and Treatment of Cancer Quality of Life Questionnaire Oral Health Module
OHIP: Oral Health Impact Profile
GOHAI: Geriatric Oral Health Assessment Index
OHIDL: Oral Health Impact on Daily Living
DSQ: Dental Satisfaction Questionnaire
OHAI: Oral Hygiene Assessment Instrument
OIDP: Oral Impacts on Daily Performance
LORQ: Liverpool Oral Rehabilitation Questionnaire
QoLDAS-9: Oral Aesthetic-related quality of life
PQL: Prosthetic Quality of Life
PRISMA: Preferred Reporting Items for Systematic Reviews and Meta-analysis
PRO: patient-reported outcomes
PROQOLID: Patient-Reported Outcome and Quality of Life Instruments Database
IPQ-RDE: Illness Perception Questionnaire Revised for Dental Use in Older/Elder Adults
