# Supplementary material for: Evaluating conceptual model measurement and psychometric properties of Oral health-related quality of life instruments available for older adults: a systematic review
Source: Health Qual Life Outcomes. 2024 Jan 13;22:5. doi: 10.1186/s12955-023-02218-7 (PMC10787424; doi:10.1186/s12955-023-02218-7)
Supplement: Supplementary file 2 — Additional file 2. [file 12955_2023_2218_MOESM2_ESM.docx]

**Evaluating oral health related-quality of life in older adults: a systematic, standardized comparison of available instruments**

Naira Figueiredo Deana MSc^a,b^, Yolanda Pardo,PhD^c,d,e^, Montse Ferrer PhD^c,d,e^, Gerardo Espinoza-Espinoza DDS, MSc,^b,f^ Olatz Garin,PhD^d,e,g^ Patricia Muñoz-Millán, DDS, MSc^1,2^ , Claudia Atala-Acevedo, DDS, MSc^a,b^ Àngels Pont,PhD^d,e^ Margarita Cancino,PhD^h,i^, Carlos Zaror DDS, MSc, PhD^a,b^

^a^ Department of Pediatric Dentistry and Orthodontics, Faculty of Dentistry, Universidad de La Frontera, Temuco, Chile.

^b^ Center for Research in Epidemiology, Economics and Oral Public Health (CIEESPO), Faculty of Dentistry, Universidad de La Frontera, Temuco, Chile.

^c^ Universitat Autònoma de Barcelona, Barcelona, Spain

^d^ Health Services Research Group, IMIM (Hospital del Mar Medical Research Institute), Barcelona, Spain

^e^ CIBER Epidemiología y Salud Pública (CIBERESP), Barcelona, Spain

^f^ Department of Public Health, Faculty of Medicine, Universidad de La Frontera.

^g^ Universitat Pompeu Fabra (UPF), Barcelona, Spain

^h^ Department of Psychology, Universidad de La Frontera, Temuco, Chile

^i^ Laboratory of cognition, Aging and Health, Universidad de La Frontera, Temuco, Chile

Corresponding author:

C Zaror DDS, MSC, PhD [carlos.zaror@ufrontera.cl](mailto:carlos.zaror@ufrontera.cl)

Department of Pediatric Dentistry and Orthodontics, Faculty of Dentistry, Universidad de La Frontera, Temuco, Chile.

Manuel Monnt #112, Temuco, Chile

Y Pardo PhD, MD [ypardo@imim.es](mailto:ypardo@imim.es)

Health Services Research Group, IMIM (Hospital del Mar Medical Research Institute), Barcelona, Spain.

**Online Resource 2: Search strategy on MEDLINE**

(((((((((((((((((questionnaire*) OR instrument*) OR scale*) OR index*) OR survey*) OR profile*) OR batter*) OR inventor*) OR measur*) OR rating*) OR item*) OR ("Surveys and Questionnaires"[Mesh])))) AND (((((((((((((((("Validation Studies" [Publication Type]) OR Psychometrics) OR "Psychometrics"[Mesh]) OR Cross-Cultural Comparison) OR "Cross-Cultural Comparison"[Mesh]) OR psychometric*) OR feasibility) OR reliability) OR validation) OR validity) OR develop*) OR translat*) OR Chronbach*) OR "Factor Analysis, Statistical"[Mesh]) OR (cross-cultural AND adaptation)))) AND ((((((((((("Quality of Life"[Mesh]) OR Quality of life) OR HRQoL) OR OHRQoL) OR QoL) OR QALY) OR utility) OR disability) OR "Disability Evaluation"[Mesh]) OR "Quality-Adjusted Life Years"[Mesh]))) AND (((((((((((((((((((((((((((((“Oral Health”[MeSH]) OR “Dental Caries”[MeSH]) OR oral[tiab] OR"Periodontitis"[Mesh]) OR caries[tiab]) OR orofacial[tiab]) OR dental[tiab]) OR periodontitis[tiab]) OR gingivitis[tiab]) OR "Gingivitis"[Mesh]) OR traumatic dental injur*) OR maxillofacial[tiab]) OR temporomandibular[tiab]) OR tooth[tiab]) OR teeth[tiab]) OR "Periodontal Diseases"[Mesh]) OR "Tooth"[Mesh]) OR "Maxillofacial Injuries"[Mesh]) OR "Dentofacial Deformities"[Mesh]) OR oral cancer[tiab]) OR "Mouth Neoplasms"[Mesh]) OR malocclusion[tiab]) OR "Malocclusion"[Mesh] OR "Pathology, Oral"[Mesh]))))))))) AND (((((((((((((((old*) OR elderly) OR senior) OR ancient*) OR retire*) OR senile) OR senescen*) OR aged))) AND (((((residen*) OR citizen*) OR person*) OR people) OR adult*))) OR "Aged"[Mesh]) OR ("Aged, 80 and over"[Mesh])) OR geriatric)
